# Supplementary material for: Structural basis for capsid recruitment and coat formation during HSV-1 nuclear egress
Source: eLife. 2020 Jun 24;9:e56627. doi: 10.7554/eLife.56627 (PMC7340501; doi:10.7554/eLife.56627)
Supplement: Supplementary file 1. — UL25∆44 Q72A/NEC particles used for cryoET averaging. List of primers used for cloning procedures described in Materials and methods. [file elife-56627-supp1.docx]

**Supplementary File 1**

**Structural basis for capsid recruitment and coat formation during HSV-1 nuclear egress**

Elizabeth B. Draganova^1^, Jiayan Zhang^2,3,4^, Z. Hong Zhou^2,3,4^, and Ekaterina E. Heldwein^1,#^

^1^Department of Molecular Biology and Microbiology, Tufts University School of Medicine, Boston, MA 02111, USA

^2^Department of Microbiology, Immunology & Molecular Genetics, University of California, Los Angeles (UCLA), Los Angeles, California, 90095, USA

^3^Molecular Biology Institute, UCLA, Los Angeles, California, 90095, USA

^4^California NanoSystems Institute, UCLA, Los Angeles, California, 90095, USA

Supplementary File 1 contains Supplementary Tables 1 and 2

| **Vesicle** | **Vesicle Radius (nm)** | **Vesicle Area*^a^* (nm^2^)** | **Particles forming**  **5-pointed stars*^b,c^*** | **Particles forming**  **5-pointed stars (picked for analysis)** | **Area of particles forming 5-pointed stars*^d^* (nm^2^)** | **Ratio of particles forming 5-pointed stars/vesicle (%)** |
| --- | --- | --- | --- | --- | --- | --- |
| 1 | 105 | 34,521 | 233 | 155 | 7,626 | 22 |
| 2 | 263 | 217,317 | 479 | 319 | 15,695 | 7 |
| 3 | 227 | 161,838 | 458 | 305 | 15,006 | 9 |
| 4 | 317 | 315,940 | 450 | 300 | 14,760 | 5 |
| 5 | 318 | 317,977 | 450 | 300 | 14,760 | 5 |
| 6 | 202 | 128,650 | 462 | 308 | 15,154 | 12 |
| 7 | 399 | 500,418 | 459 | 306 | 15,055 | 3 |
| 8 | 187 | 109,794 | 195 | 130 | 6,396 | 6 |
| 9 | 249 | 195,456 | 453 | 302 | 14,858 | 8 |
| 10 | 267 | 223,775 | 453 | 302 | 14,858 | 7 |
| **Average** | **253** | **220,569** | **409** | **273** | **13,417** | **8** |

**Supplementary Table 1. UL25∆44 Q72A/NEC particles used for cryoET averaging.** The estimated vesicle surface coverage (%) by particles picked for cryoET data processing was calculated using certain assumptions, as indicated within the table.

*^a^* We assume all vesicles are spherical; Area = π * r * r; r = radius

*^b^* 90% of particles picked from tomograms are forming 5-pointed star shaped density (based on the cross-correlation coefficient (CCC) value)

*^c^* 60% of particles forming 5-pointed star shaped density were picked from tomograms

*^d^* We assume the area of each 5-pointed star = $\frac{1}{4}\sqrt{5(5+2\sqrt{5)}}*{9.8}^{2}=164 {nm}^{2}$ (side of UL25 pentagon = 9.8 nm; see Figure 5)

**Supplementary Table 2.** **List of primers used for cloning procedures described in Materials and Methods.** All primers are listed in the 5’-3’ direction. Restriction sites are underlined and mutations are bolded.

| **Primer Name** | **Primer Sequence (5’-3’)** | **Restriction Site** |
| --- | --- | --- |
| UL25Δ44 fwd (JB133) | aaaaaaggatcccgtgaaaccgcagcagaacagg | BamHI |
| UL25Δ50 fwd (ED034A) | aaaaaaggatcccaggttgttgttctgcaggcacagcgt | BamHI |
| UL25Δ73 fwd (JB238) | aaaaaaggatccgccgaactgccggttgatattg | BamHI |
| UL25 rev (JB134) | aaaaaactcgagttattacactgcgctcagatactgagg | XhoI |
| UL25Δ44 Q72A (ED006) | aatgcagcaatg**gcg**gcagcc | Site-directed |
| UL25Δ44 Q72A (ED007) | ggctgc**cgc**cattgctgcatt | Site-directed |
| eGFP fwd (ED010) | aaaaaaggatccgtgagcaagggcgaggagctg | BamHI |
| eGFP rev (ED049) | aaaaaaggatcccttgtacagctcgtccatgccgagagtg | BamHI |
| UL31 CBM fwd (JB208) | ccgtgtcggccgca**gcc**atttat**gctgca**atgagg**gcc**atcagcttcgacggg | SOE |
| UL31 CBM rev (JB209) | cccgtcgaagctgat**ggc**cctcat**tgcagc**ataaat**ggc**tgcggccgacacgg | SOE |
