# Supplementary material for: Structural basis for capsid recruitment and coat formation during HSV-1 nuclear egress
Source: eLife. 2020 Jun 24;9:e56627. doi: 10.7554/eLife.56627 (PMC7340501; doi:10.7554/eLife.56627)
Supplement: Transparent reporting form [file elife-56627-transrepform.docx]

***eLife’s* transparent reporting form**

We encourage authors to provide detailed information *within their submission* to facilitate the interpretation and replication of experiments. Authors can upload supporting documentation to indicate the use of appropriate reporting guidelines for health-related research (see [EQUATOR Network](http://www.equator-network.org/%20)), life science research (see the [BioSharing Information Resource](https://biosharing.org/)), or the [ARRIVE guidelines](http://www.plosbiology.org/article/info:doi/10.1371/journal.pbio.1000412) for reporting work involving animal research. Where applicable, authors should refer to any relevant reporting standards documents in this form.

If you have any questions, please consult our Journal Policies and/or contact us: [editorial@elifesciences.org](mailto:editorial@elifesciences.org).

**Sample-size estimation**

- You should state whether an appropriate sample size was computed when the study was being designed
- You should state the statistical method of sample size computation and any required assumptions
- If no explicit power analysis was used, you should describe how you decided what sample (replicate) size (number) to use

Please outline where this information can be found within the submission (e.g., sections or figure legends), or explain why this information doesn’t apply to your submission:

No sample size calculation was performed. Sample sizes were chosen based on standard practices for comparable experimental setups. Sample sizes were sufficient as judged by the high reproducibility of the mean values reflected in the small standard error values of the mean.

**Replicates**

- You should report how often each experiment was performed
- You should include a definition of biological versus technical replication
- The data obtained should be provided and sufficient information should be provided to indicate the number of independent biological and/or technical replicates
- If you encountered any outliers, you should describe how these were handled
- Criteria for exclusion/inclusion of data should be clearly stated
- High-throughput sequence data should be uploaded before submission, with a private link for reviewers provided (these are available from both GEO and ArrayExpress)

Please outline where this information can be found within the submission (e.g., sections or figure legends), or explain why this information doesn’t apply to your submission:

The amount of biological and technical replicates is provided for applicable experiments in the following figure legends: Fig. 1, 2, and 3.

The definition of biological and technical replicates is provided in the Materials and Methods section within the co-sedimentation assay sub-section (line 590).

The raw data values for experiments presented in Fig. 1, 2, and 3 are provided in Supplementary Table S2. Individual biological and technical replicates are color-coded. Replicates are also identifiable within the figures themselves.

Outliers were not encountered and data were not excluded.

**Statistical reporting**

- Statistical analysis methods should be described and justified
- Raw data should be presented in figures whenever informative to do so (typically when N per group is less than 10)
- For each experiment, you should identify the statistical tests used, exact values of N, definitions of center, methods of multiple test correction, and dispersion and precision measures (e.g., mean, median, SD, SEM, confidence intervals; and, for the major substantive results, a measure of effect size (e.g., Pearson's r, Cohen's d)
- Report exact p-values wherever possible alongside the summary statistics and 95% confidence intervals. These should be reported for all key questions and not only when the p-value is less than 0.05.

Please outline where this information can be found within the submission (e.g., sections or figure legends), or explain why this information doesn’t apply to your submission:

The statistical analysis methods along with values of N, and definition of error bars (SEM) for all applicable experiments (Fig. 1, 2, and 3) are presented in the figure legends and within the Material and Methods section.

Individual data points are presented in each applicable figure (Fig. 1, 2, and 3) and all raw data values are within Supplementary Table S2.

p-values are listed in figure legends for each applicable figure (Fig. 1, 2 and 3).

(For large datasets, or papers with a very large number of statistical tests, you may upload a single table file with tests, Ns, etc., with reference to sections in the manuscript.)

**Group allocation**

- Indicate how samples were allocated into experimental groups (in the case of clinical studies, please specify allocation to treatment method); if randomization was used, please also state if restricted randomization was applied
- Indicate if masking was used during group allocation, data collection and/or data analysis

Please outline where this information can be found within the submission (e.g., sections or figure legends), or explain why this information doesn’t apply to your submission:

Sample blinding was not performed. Allocation for either co-sedimentation or budding assays was not at random. The covariate background signal was subtracted from all raw data values before data analysis. This is indicated in the Materials and Methods section.

Masking was not used at any point in this analysis.

**Additional data files (“source data”)**

- We encourage you to upload relevant additional data files, such as numerical data that are represented as a graph in a figure, or as a summary table
- Where provided, these should be in the most useful format, and they can be uploaded as “Source data” files linked to a main figure or table
- Include model definition files including the full list of parameters used
- Include code used for data analysis (e.g., R, MatLab)
- Avoid stating that data files are “available upon request”

Please indicate the figures or tables for which source data files have been provided:

Fig. 1, 2, and 3
